# Draft genome sequences of three bacteria with antimicrobial activity isolated from pond sediment on a university campus

**DOI:** 10.1128/mra.00053-26

**Published:** 2026-05-29

**Authors:** Holly Moffett, Naomi Inthirath, Jessica Mojica, Delia Moreno, Marcos Martinez, Taylor Hedley, Arielle Names, Cassandra M. Haakma, Priscilla J. Garcia, Ally Watkins, Tricia A. Van Laar

**Affiliations:** 1 Department of Biological Sciences, California State University, Stanislaus 14674, Turlock, California, USA; Loyola University Chicago, Chicago, Illinois, USA

**Keywords:** *Pseudomonas*, *Priestia*, antimicrobial activity

## Abstract

We isolated three organisms from pond sediment with activity against *Bacillus subtilis* and/or *Staphylococcus aureus*. Isolates were identified as *Priestia megaterium* (MBIO4670.03), *Pseudomonas aeruginosa* (MBIO4670.02), and a *Pseudomonas* sp. in the *putida* group (MBIO4670.08). Each isolate contains one or more biosynthetic gene clusters potentially contributing to the observed antimicrobial phenotype.

## ANNOUNCEMENT

Our Microbial Ecology course at California State University, Stanislaus, isolated bacteria with antimicrobial activity from pond sediment at an approximate depth of 10 cm (37.522444 N 120.852833 W). Each group resuspended 1 g of sediment from the selected location in PBS and plated 10-fold serial dilutions of on tryptic soy agar (TSA) supplemented with 50 μg/μL cycloheximide for incubation at 37°C overnight (1). Isolated colonies were patched on TSA with lawns of various bacteria including *B. subtilis* (ATCC 23857) and *S. aureus* (ATCC 25923) and incubated at 37°C overnight. Colonies inhibiting growth of a test bacterium were grown in TSB overnight at 37°C with shaking. DNA was extracted utilizing Promega Wizard Genomic DNA Purification Kit according to manufacturer’s instructions for Gram-positive bacteria. The quality and quantity of purified DNA was measured using a NanoDrop Spectrophotometer. DNA was sent to SeqCenter (Pittsburgh, PA) for library preparation and sequencing. Libraries were prepared using tagmentation-based and PCR-based Illumina DNA Prep kit and custom IDT 10 bp unique dual indices with a target insert size of 280 bp prior to sequencing on an Illumina NovaSeq X Plus, producing 2 × 151 bp paired-end reads. All software described were used according to default parameters. Demultiplexing, quality control, and adapter trimming were performed using the proprietary Illumina software bcl-convert (v4.2.4). We used Fastp v1.0.1 (2) for additional filtering and trimming of low-quality reads. We assembled our genomes using SPAdes v4.1.0 (3) and assessed assembly quality using QUAST v5.3.0 (4). At this point, we determined that one genome was significantly contaminated, so we used Kraken2 (5) Galaxy Version 2.17.1 + galaxy0 with the PlusPF-16 database to identify the predominant species (*Ps. aeruginosa*) and KrakenTools Galaxy Version 1.2.1+galaxy0 (--fastq_output, --include_children, --taxid 135621) to extract reads belonging to Pseudomonadaceae from the trimmed reads produced by Fastp. The final assembly of the *Ps. aeruginosa* genome was performed as described above on the reads filtered by Kraken. Assembled reads were screened, and any final contaminates removed using the NCBI Foreign Contamination Screen (6) workflow implemented in Galaxy (--div Bacteria, --min-seq-len 500, taxonomy Prokaryotes). Genome completeness and contamination were assessed using CheckM2 (7) Galaxy Version 1.10 + galaxy0. We used the Prokaryotic Genome Annotation Pipeline (PGAP v 6.10 [8]) to annotate all genomes. Table 1 contains the detailed statistics for each assembly.

**TABLE 1:** Genomic features and accession information for all isolates

	*Priestia megaterium* (MBIO4670.03)	*Pseudomonas aeruginosa* (MBIO4670.02)	*Pseudomonas* sp. *(putida)* (MBIO4670.08)
Activity against	*B. subtilis*	*B. subtilis*	*B. subtilis, S. aureus*
Raw number of bases	550,854,433	679,010,041	582,363,999
Raw number of reads	3,776,520	4,624,628	3,932,984
Trimmed number of bases	547,906,937	669,672,790	573,587,082
Trimmed number of reads	3,756,670	4,562,474	3,875,228
Filtered number of bases		600,672,530	
Filtered number of reads		4,071,418	
Genome length (bp)	5,989,506	6,542,757	5,857,155
Average fold coverage (×)	91.48	102.35	97.93
GC content (%)	37.36	66.25	62.01
No. of contigs	119	142	130
Contig N50 (bp)	373,448	198,679	85,370
Contig L50	5	9	21
No. of CDS	6,163	6,153	5,270
No. of rRNAs	9	3	5
No. of tRNAs	46	51	43
CheckM2 completeness	100	100	100
CheckM2 contamination	0.6	0.02	0.47
Biosample accession	SAMN53653665	SAMN53671806	SAMN53703027
SRA accession	SRR36301676	SRR36309611	SRR36334374
Genome accession	JBSRRE000000000	JBSVJO000000000	JBSVDF000000000
Version reported	JBSRRE020000000	JBSVJO020000000	JBSVDF020000000

**Fig 1:**
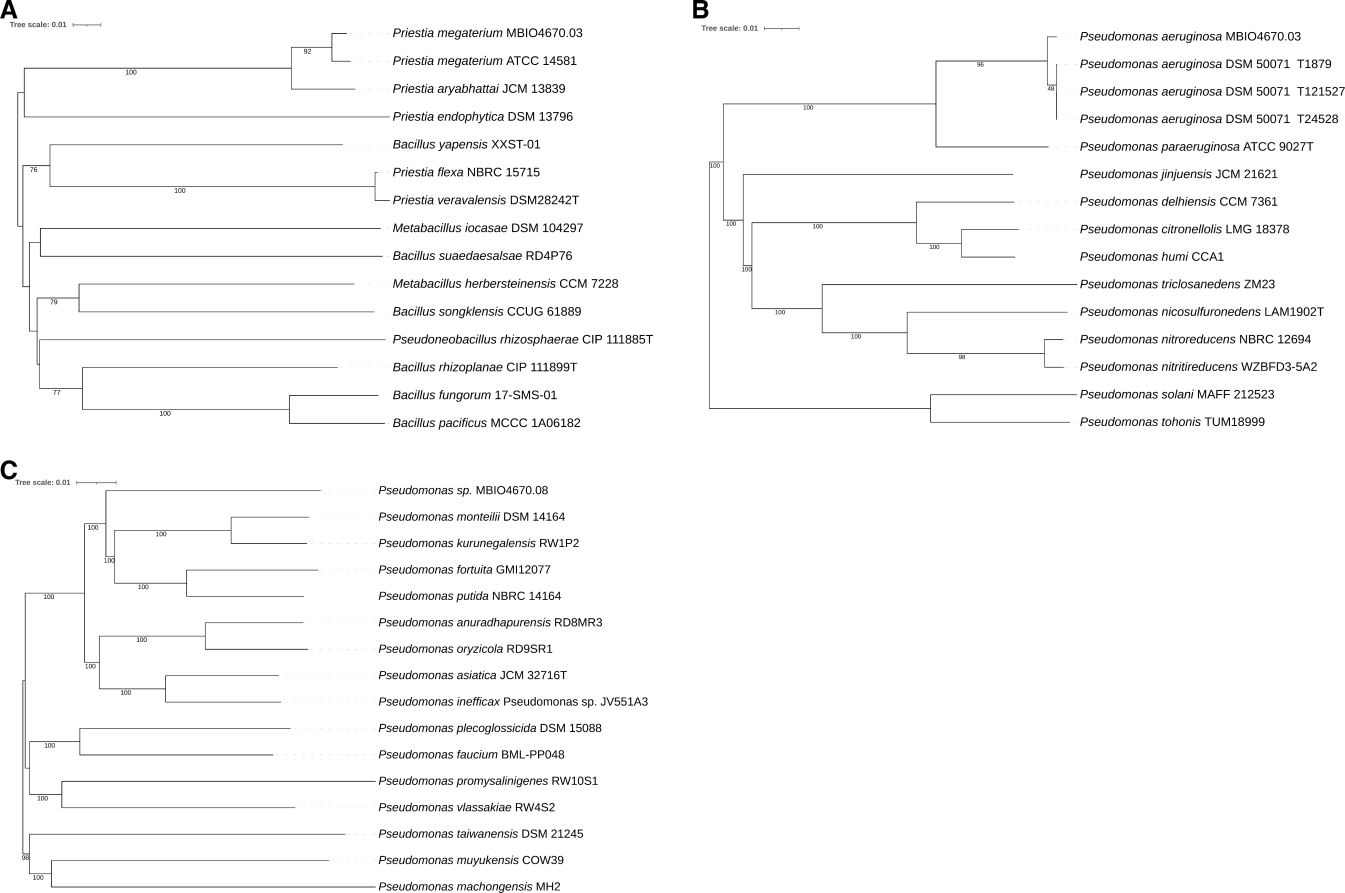
Tree inferred with FastME 2.1.6.1 (15) from Genome BLAST distance phylogeny (GBDP) distances calculated from genome sequences. The branch lengths are scaled using GBDP distance formula *d_5_*. Numbers are GBDP pseudo-bootstrap support values >60% from 100 replications, with an average branch support of 89.8% (*Priestia megaterium*; **A**), 96.8% (*Pseudomonas aeruginosa*; **B**), and 60.3% (*Pseudomonas* sp. *putida* group; **C**). The tree was rooted at the midpoint (16).

Phylogenetic analysis using the Type Strain Genome Server (9) and Interactive Tree of Life v. 7 (10) supported the identification of *Pr. megaterium* and *Ps. aeruginosa* and placed the third isolate with the *putida* group with 62.6% similarity to *Ps. putida* based on dDDH_4_ values (Fig. 1). antiSMASH 8.0 (11) identified candidate biosynthetic gene clusters possibility contributing to the antimicrobial phenotype. *Pr. megaterium* contained a paeninodin-like lassopeptide biosynthetic gene cluster. Some lassopeptides exhibit antimicrobial activity (12). *Ps. aeruginosa* contained genes for the production of hydrogen cyanide, phenazines, and pyoluteorin which is produced by fluorescent *Pseudomonas* and inhibits *B. subtilis* (13). *Ps*. sp. (*putida*) encoded a putative azole-containing ribosomally synthesized and post-translationally modified peptide (RiPP) cluster. Azole-containing RiPPs frequently have antimicrobial activity (14).

## Data Availability

All data are available from BioProject PRJNA1373347. The SRA and genome assembly accession numbers are provided in Table 1.
